# Cycling for a Sustainable Touristic Mobility: A Preliminary Study in an Urban Area of Italy

**DOI:** 10.3390/ijerph182413375

**Published:** 2021-12-19

**Authors:** Gabriella Mazzulla, Maria Grazia Bellizzi, Laura Eboli, Carmen Forciniti

**Affiliations:** Department of Civil Engineering, University of Calabria, 87036 Rende, Italy; mariagrazia.bellizzi@unical.it

**Keywords:** bike lane, cycling, sustainable touristic mobility, service quality, public health

## Abstract

This study wants to give a contribution for the investigation of sustainable mobility with positive consequences on public health implementing policies starting from cyclists’ perceptions. Data were collected by interviewing cyclists along three bike lanes of an urban area of southern Italy through a face-to-face survey. The survey was conducted in Autumn 2019, interviewing a sample of 129 cyclists. In order to identify the critical aspects of the bike paths, both an importance-performance analysis (IPA) and a gap-IPA were performed. The average values of the cyclists’ perceptions of each aspect have been considered as performance values. The importance values have been obtained by performing a principal component analysis (PCA), which was helpful also for better defining the service quality phenomenon. From the PCA, six latent constructs can be identified as: “Physical Nuisance”; “Non-physical Nuisance”; “Physical Comfort”; “Non-physical Comfort”; “Protection”; and “Ambience”. The results of Gap-IPA confirmed that the criticalities of the bike paths relate to the degree of protection in relation to accidents, and to the degree of nuisance caused by pollution and opposing pedestrians along the path. Based on the conducted analyses, sustainable tourism implementing policies should be oriented in solving the emerged criticalities of the existing bike paths. The results of Gap-IPA are very intuitive and can certainly be helpful for identifying the most convenient strategies.

## 1. Introduction

As recognized worldwide, the tourism industry plays a key role in the economic, social, and cultural development of an area [1]. However, together with its benefits, it produced also several environmental impacts [2].

The need to incorporate the paradigm of sustainability into tourism has been largely discussed in the tourism industry and has also emerged as an important field of the academic research [3] for both professionals and policy makers. Today, there is no unanimous consensus on the concept of sustainable tourism. However, the World Tourism Organization universally defines it as “tourism that takes full account of its current and future economic, social, and environmental impacts, addressing the needs of visitors, the industry, the environment, and host communities” [4]. They point out on the concept that a sustainable tourism cannot exist without considering together the benefits and the environmental impacts this industry involves with respect to tourist destinations.

Transport modes play an important role in tourism development, but a considerable part of the environmental impacts of the tourism industry is caused by transports too. There is a close relationship between sustainable transport and sustainable tourism; a transport system can be considered sustainable if it is accessible, safe, environmentally friendly, and affordable [5,6]. On the contrary, when great emissions of greenhouse gases or a massive consumption of energy is a consequence of the used transport mode, then the transport systems can be considered as not sustainable [7,8,9].

As recognized also by [6], when long distances are needed for reaching touristic locations and people desire quickly accessing to tourist destination, less sustainable transport modes were used (e.g., air transport, private car, coach). The only exception is the railways, which represents a good compromise especially from medium to long distances. In urban areas, where the distances for reaching touristic attractions are quite short, very sustainable transport modes can be used such as walking and cycling. Distances covered by using active mobility modes produce environmental impacts significantly reduced. Consequently, an effort must be made for promoting, for example, non-motorized transport systems [10]. The potential of cycling for implementing a sustainable tourism has been widely recognized in both academic and policy circles [11]. In fact, substantial public investments in bike infrastructure are being made in many parts of the world, largely motivated by the goal of improving public health and increasing the sustainability of the transportation system [12].

Promoting cycling can help to achieve a sustainable tourism system with important environmental, social, and public health benefits. In cities where the levels of pollution are relevant, the health benefits of cycling have been documented as being greater than the risks for exposure to low air quality [13]. In urban areas, cycling is also a cheap form of transport and sometimes can be faster than other transport modes, allowing cyclists to avoid traffic bottlenecks [14]. Thigpen [15] thinks that life changes can affect individuals’ travel behavior, and incentivizing bike use can lead to a shift in perceptions and attitudes about cycling, with a positive feedback cycle between greater bicycling attitudes and skills and increased bicycle use.

Two main goals can be obtained by betting on bike infrastructures. The first one consists in contributing to a sustainable transportation system by promoting urban cycling as an alternative transport mode for making the various activities in an urban area and reaching activity destinations. In such a context, all of the tourists, both cyclists and other users, can benefit from the advantages of an environment offering more space for making different activities, less pollution and noise levels, and greater safety. The second goal that can be reached by promoting cycling culture and increasing the bike infrastructures represents a direct benefit for the tourists who want to move by bike since they increase their physical activity and wellbeing. This is the so-called bicycle tourism. Cycling tourists have become an increasingly common sight in many European cities such as Berlin and Copenhagen [9]. There are several categories of bicycle tourists: the proper bicycle tourists, for whom cycling is an important reason for travelling (for sports, long distance journeys or for taking multiple excursions), and a less visible category, called holiday cyclists, for whom cycling forms part of the holiday experience but it is not their main focus [12]. Definitively, the two concepts of urban cycling and bicycle tourism can be conveniently integrated among them, and the development of urban cycling and bicycle tourism may be viewed as a case of best practice [9].

Investing in bike infrastructure has been considered an effective measure to increase cycling in cities and, consequently, to promoting the use of bicycle both for tourists and citizens. An extensive literature shows a strong connection between bike infrastructure and cycling as a mode of transportation [14,16,17,18].

In order to opportunely address planning policies and the investments to be made, the knowledge of the cyclists’ point of view becomes fundamental. Griswold et al. [19] pointed out that cyclist behavior and bicycle user experience can help to guide both design and investment considerations. Many factors can be relevant in individuals’ decision to bicycle. As an example, the role of safety in encouraging or discouraging individuals’ decision has been the focus of a several studies (e.g., [20]). Ma and Dill [21] found that the propensity of cycling and frequency of trips by bike depend on perceptions of the built environment, whereas [22] established that bike facilities characteristics as trails, lanes, or traffic volume affect individual preferences for different cycling environments. Additionally factors concerning individual attitudes and perceptions have an effect on individuals’ travel behavior and on their decision to start cycling (e.g., [23]).

Some authors investigated cyclists’ perceptions with the final aim of finding important information useful for promoting cycling culture and improving the quality of bike infrastructures. As an example, Damant-Sirois et al. [24] proposed different strategies to each type of cyclist starting from their characteristics and behavior. Rodriguez-Valencia et al. [11] explored the increase in bicycle commuting in Bogota (Colombia) by examining the underlying factor responsible for this, which is the motivation of people using the bike for transport. Specifically, through a survey administered to cyclists in Bogota, they analyzed the personal attitudes, preferences, travel behavior, social influences, and motivations of individuals who shifted to the bike. Through a survey made to individuals from Quito (Ecuador), Caicedo et al. [25] evaluated the perception of barriers to bike use and discovered the importance of certain variables such as a lack of bike infrastructure. Their study highlights that people’s needs are a crucial point in developing strategies to promote bike use, such that mobility services can be capable of retaining existing users and attract new ones (especially from motorized modes). Other authors discovered that psycho-social and attitudinal factors that make the bike eligible as a modal alternative have an important role in predicting the intention to cycle [26,27,28]. Fernández-Heredia et al. [26] conducted a survey for collecting cyclists’ perceptions and understanding their behavior in order to determine the appropriate actions to encourage bike use. They planned an in-depth investigation of cyclists’ perceptions using a large university survey conducted in Madrid (Spain). Heinen et al. [27] proposed a study where the influence on the mode choice of commuters’ attitudes toward the benefits of travel by bike was analyzed; the study was conducted in Delft and Zwolle (The Netherlands). Specifically, convenience, low cost, and health benefits were considered. Cepeda-Zorrilla et al. [28] conducted a study addressed to commuters in Mexico City (Mexico) and learned that people are conditioned by many barriers when choosing to cycle in a metropolitan area.

By following this research orientation, we propose in this paper an analysis of the perceptions of a sample of cyclists using bike paths in an urban area. Specifically, an importance-performance analysis (IPA) and a gap-IPA were performed on the basis of the collected data, with the aim to discover the criticalities of the bike paths and understanding the most important aspects for the users. From a methodological point-of-view, we think to give a contribution by introducing a very intuitive tools for capturing the cyclist perceptions and for identifying the most convenient strategies for promoting urban cycling and bicycle tourism. In the following, we report a section describing the research methodology consisting in the method of collecting the data and the method for analyzing the data (i.e., IPA and gap-IPA). The successive section shows the application of IPA and gap-IPA to the data collected by the experimental survey. Finally, the main conclusions are reported.

## 2. Research Methodology

**Figure 1:**
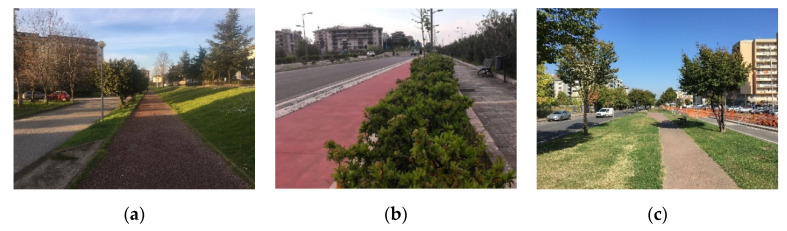
Present state of cycle paths: (**a**) Villaggio Europa, (**b**) Viale Principe, (**c**) Viale Mancini.

The data of this study were collected through a survey conducted in an urban area of southern Italy. Specifically, the urban area is composed of the two towns of Cosenza and Rende, which are so close that one can be considered an extension of the other. The population of the urban area reaches up to 120,000 inhabitants, thanks also to the presence of the University, which attracts roughly 30,000 students and 2000 employees, among teachers, technical, and administrative staff. In addition, the urban area is full of history and boasts several tourists’ attractions such as churches, museums, buildings with historical relevance, and natural areas. From a geographical point of view, the urban area of Cosenza and Rende is prevalently flat and characterized by a mild climate. For all the above reasons, the urban area is particularly suitable to the use of bike. As a matter of fact, there are several bike paths developing in the area. However, for implementing the sustainable tourism based on cycling there are two main issues to be solved: (1) the use of bike is still poor in the urban area; (2) the bike paths are not strictly connected to the main tourist attraction. This last is not object of this study, whose aim is instead identifying the underlying causes of the first issue. According to this, it was decided to investigate directly on the cyclists’ perceptions about existing bike paths in order to identify their strengths and weaknesses and, as a consequence, to give a contribution for sustainable tourism implementing policies. Three cycle paths were selected, these being considered the most important ones of the urban area. Although they are very close, the three cycle paths are not connected among them. Two bike paths (Figure 1a,b) develop within the municipality of Rende, and one within the municipality of Cosenza. The first one (Figure 1a) runs around the social housing area of “Villaggio Europa” for about 3 km. The second bike path (Figure 1b) develops for about 5 km alongside the two roadways of “Viale Principe”. Finally, the third bike path is roughly 3 km and extends alongside “Viale Mancini”. As infrastructure types, the three bike paths are very similar. They are characterized by two-way bikeways with red paving, separated from the motorized traffic and surrounded by green areas.

The interviews for collecting data were carried out face-to-face and addressed to randomly stopped cyclists. The questionnaire used is a paper questionnaire deriving from an ongoing research project of the Universidad de los Andes (Bogotà, Colombia). The questionnaire aimed to investigate on cyclists’ perceptions about different aspects of the bike path. Specifically, the perceptions were requested in terms of level of comfort, degree of nuisance and degree of protection. In all cases, the evaluation scale is a 11-point Likert scale ranging from 0 to 10. For the levels of comfort, 0 corresponds to “totally uncomfortable” and 10 to “totally comfortable”. Regarding the degrees of nuisance, the scale ranges from “absence of nuisance” to “high degree of nuisance”. Finally, the degrees of protection vary from “absence of protection” to “high degree of protection”. The questionnaire ends with some questions for collecting information about the trip (origin and destination, purpose, estimated time for reaching the destination, frequency of trip) and about the interviewee (e.g., gender, age, employment and marital status).

The interviews took place from September to October 2019 and a sample of 129 interviewed cyclists was obtained. In Table 1 the main sample’s socio-demographic and trip characteristics are reported. Interviewed people are prevalently males (55.8%) than females, and most of them are between 31 and 50. The sample is composed mostly of employee (41.1%), freelancer workers (20.2%), and students (23.3.%). More than 45% of cyclists declared to use the bike path once a week, and 23.3% rarely. This result confirms the poor attitude for cycling in the area. Finally, in terms of trip purpose, interviewed people used the bike path prevalently for sport activities (75.2%).

In order to identify the critical aspects of the bike paths, both an IPA and a gap-IPA were performed. The IPA is a well-known technique in the literature, proposed initially by Martilla and James [29]. It is based on two dimensions, namely performance and importance, graphically depicted along the *x*-axis and the *y*-axis respectively. The means of performance and importance divide the chart into four quadrants. The first quadrant, where both performance and importance values are higher than the means, contains the strengths aspects. Otherwise, the second quadrant contains the major weaknesses since the performance is low and importance is high. The third quadrant contains the minor weaknesses since both the performance and the importance are lower than the means. Finally, the fourth quadrant contains the minor strengths since the performance is high and importance is low. The gap-IPA is a simplification of IPA proposed in [30] where it proved to be very useful in highlighting the critical issues of the analyzed service. With this technique, the gap between importance and performance is graphically reported on a circular graph composed only of two sectors. The external one contains the “criticalities” of the service, or the aspects whose importance is higher than performance. Otherwise, the “non-priorities” sector contains the aspects whose performance is higher than importance. In this study, the average values of the cyclists’ perceptions of each aspect of the bike paths have been considered as performance values. The importance values have been obtained by performing a PCA, which was helpful also for better defining the service quality phenomenon of the bike paths.

## 3. Analysis and Findings

In Table 2, the average values of the cyclists’ perceptions expressed for each aspect of the bike paths are reported. From the obtained results, it can be observed that all of the levels of comfort vary between 6 and 7, while most of the degrees of nuisance and protection vary between 5 and 6. Therefore, bike paths performances in terms of comfort are slightly above the middle of the evaluation scale. Otherwise, the performances in terms of nuisance and protection are slightly below the respective evaluation scales. As a consequence, it can be said that the bike paths are quite comfortable for cyclists, especially in terms of interaction with other bike along the routes, with the environment, and with landscapes surrounding the paths. With respect to the degrees of nuisance, it may be helpful to point out that to a low degree of nuisance corresponds to a low rating. Therefore, the main causes of nuisance are the traffic speed and volume on the roadways alongside the bike paths, pollution, and noise. Since for the degree of protection the absence of protection corresponds to the lowest rating of the evaluation scale, it emerges that the bike paths are poorly protected from all the investigated aspects. 

Then, a PCA was conducted in order to better define the cyclists’ perceptions on the bike paths. The PCA was performed using SPSS software (IBM, Armonk, NY, USA). Kaiser-Meyer-Olkin (KMO) measure of sampling adequacy is acceptable (0.795). The Barlett test of sphericity is verified (significance at 0.000), and therefore sufficient correlations exist among the variables to proceed. The method of extraction is principal components analysis and the rotation method is Varimax with Kaiser normalization. Varimax is the most popular orthogonal factor rotation method and it is preferred to other orthogonal rotation methods in achieving a simplified factor structure [31]. The total variance explained by the extracted constructs is about 74%. All of the absolute loadings are higher than 0.4, so the rule of [32] for including the item in the construct is always respected. Cronbach’s alpha for each construct is higher than 0.75, whereby the internal consistency is reliable [31]. In Table 3, the obtained results from the PCA are reported. From the PCA, six latent constructs were identified: “physical nuisance”; “protection”; “physical comfort”; “non-physical nuisance”; “non-physical comfort”, and “ambience”. The first one includes all the elements that represent a physical obstruction to the bikes’ passage on the path, or a visual clutter as well. For these reasons, the construct was named “physical nuisance”. The second construct groups all the paths’ aspects investigated by the questionnaire in terms of degree of protection. The third construct relates to the items whose presence causes physical comfort (e.g., shady areas) or discomfort (e.g., other bikes). “non-physical nuisance” and “non-physical comfort” are similar to the first and third constructs respectively, however, they relate to those aspects not properly physical. Finally, the last latent construct groups the cyclists’ perceptions about environment and landscape surrounding the bike paths. Therefore, in terms of tourism attractiveness, the “ambience” construct can be considered as the most interesting one.

As mentioned above, the obtained loadings of the PCA were used as importance values for performing IPA and gap-IPA. The two techniques were applied in order to highlight the analogies and the differences of the results, and to show the advantages of adopting gap-IPA.

For both of the applications, the values of performance and importance were normalized (Table 4) in order to express the values on the same scale. For the performance values the normalization was carried out by paying attention to the meaning of the different evaluation scales. More specifically, for those aspects whose evaluation was expressed in terms of level of comfort and degree of protection, the minimum of the scale corresponds to 0, and the maximum to 1. On the contrary, for those aspects whose evaluation was expressed in terms of degree of nuisance, the minimum of the scale corresponds to 1 and the maximum to 0. As regards the importance values, the normalization was carried out by matching 0 to the minimum value and 1 to the maximum.

**Figure 2:**
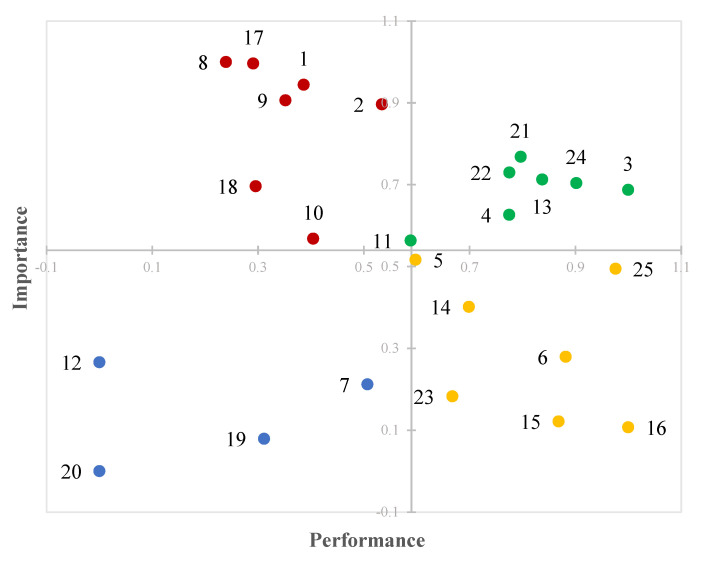
IPA: significant strengths (green), major weaknesses (red), minor weaknesses (blue), minor strengths (yellow).

By observing the Figure 2, *x*-axis (performance values) and *y*-axis (importance values) intersect at their mean values, that are 0.59 and 0.54, respectively. 

The first quadrant contains the aspects related to nuisance for scooters parked along the path and street lane width; protection from theft or robbery; comfort with shady areas; and lightening, cleanliness, and environment close to the path. Therefore, the cyclists satisfied with these aspects are considered important as well, and their quality should be maintained high in order to obtaining advantages. On the contrary, the aspects particularly in need of improvement are those of the second quadrant such as nuisance for opposing pedestrians, pedestrian flow, pollution and noise; and protection from accidents in general and from stray animals. Finally, all of the other aspects of the bike paths that fall in the last two quadrants can be considered with low priority.

**Figure 3:**
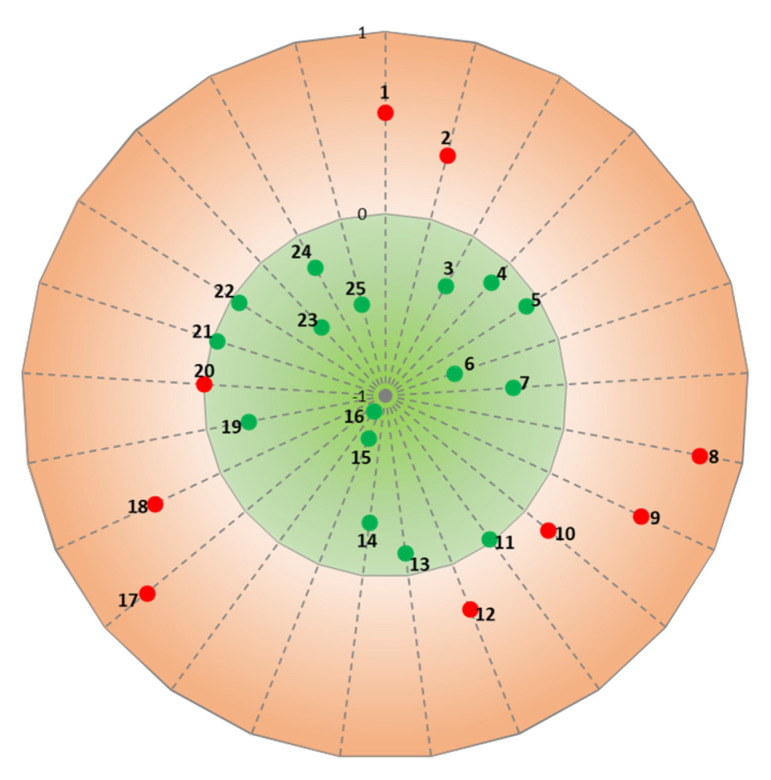
Gap-IPA: non-priorities (green), criticalities (red).

In Figure 3, the results of gap-IPA are shown. The calculated gaps of importance and performance (Table 4) are reported on the following circular graph. Most of the aspects have a gap value lower than 0, so they are located in the green sector characterizing the “non-priorities”, since performance is higher than importance. On the contrary, the “criticalities” are in the external sector and their gap is higher than 0, since their importance values are higher than their performance ones. 

The results of the gap-IPA confirmed those of the IPA, considering that the most critical issues are the aspects related to the nuisance for opposing pedestrians, pedestrian flow, pollution and noise; and protection from accidents in general and from stray animals. However, according to the gap-IPA, the protection from the weather and the nuisance for the traffic speed should also be considered as criticalities, rather than low priority aspects. Moreover, particular attention shall be paid to those aspects (e.g., comfort with lightening, cleanliness, environment close to the path and shady area) located in the green sector but near the border which could pass easily in the external sector if their performance decreases. 

## 4. Discussion

The two methods applied for analyzing the data provide comparable results. As we can see from Figure 2 and Figure 3, the output of gap-IPA is more intuitive. In addition, the results of gap-IPA seem to be more accurate since it considers only two sectors. The external one contains the “criticalities” of the service, or the aspects whose importance is higher than performance. The “non-priorities” of the service are the aspects whose performance is higher than importance and are contained in the internal sector.

According to the gap-IPA, the most critical aspects of the bike paths are the aspects related to the nuisance for opposing pedestrians, pedestrian flow, traffic speed, pollution and noise; protection from accidents in general; and protection from the weather and from stray animals. 

These results are also confirmed in the literature. The nuisance for opposing pedestrians, pedestrian flow, traffic speed, pollution, and noise can imply the separation of bike flow from pedestrian and vehicular flows. As reported by Li et al. [33], the physical separation from pedestrians produces an increase in cycling comfort. In addition, when the number of bikes along the path is relevant, the cyclists want more riding space and pay much attention to avoiding potential collisions with other bikes. 

Another important issue is the security along the path. Cyclists request a safety path. Gutierrez et al. [34] analyzed the perception of risk regarding bicycle use. They found that the incentives for using bicycles must consider the need for structural changes to diminish the latent perception of insecurity held by cyclists. Similarly, for Branion-Calles et al. [35], expanding the bicycling network and increasing the availability of bicycle infrastructure could increase the perceived safety of bicycling amongst bicyclists.

The results of our work did not determine a relevant role of the environment close to the path on the overall assessment of the quality of the cycle path. This result is also confirmed by other studies in the literature. For Ma and Dill [21], perceptions of the environment do not predict the propensity to bicycle, but only influence bicycling frequency.

## 5. Conclusions

In this paper, cyclists’ point-of-view was considered as crucial for identifying strategies for improving urban bike paths with the final aim of promoting urban cycling and bicycle tourism, which represent important policies for achieving a sustainable mobility with positive consequences on public health. Specifically, cyclists’ opinions about different bike paths were collected through a survey, and a methodology based on the use of importance and performance values expressed by the interviewed users was proposed. Specifically, an innovative method called gap-IPA, developed starting from the well-known IPA, was applied to the collected data. The advantages of gap-IPA as regards IPA consists in the most effective graphical representation of gap-IPA, which can offer a more straightforward reading of the data. IPA proposes a subdivision of the service attributes in well four quadrants, where the reader could be disoriented, while according to Gap-IPA there is a sharper division of the attributes in only two groups. Secondly, for interpreting IPA, a continuous comparison between importance and performance has to be affected, while gap-IPA adopts only one value, which is the gap between importance and performance (a more immediate approach) [30]. From these considerations, we can conclude that the results of the Gap-IPA are particularly understandable and interpretable by all the readers. The specific results emerged by the application of Gap-IPA consisted in discovering that the most critical aspects relate to the nuisance for opposing pedestrians, pedestrian flow, pollution and noise; protection from accidents in general and from stray animals; and, in addition, protection from the weather and the nuisance for the traffic speed should be considered as criticalities, rather than low priority aspects as emerged from the IPA application. All of the above-mentioned aspects represent the factors that should be improved for satisfying cyclists and offering them bike infrastructures with high levels of quality, with the final aim of promoting urban cycling and making the cycling mode attractive also for tourists. Moreover, it should be highlighted that the sample of interviewed cyclists is mostly composed of people cycling for sport reasons. Therefore, the findings emerged from the analysis can be very useful for identifying the right strategies for promoting and increasing bicycle tourism, which is mainly practiced by sportspersons. In addition, the nature of the sample, composed mainly by people cycling for sport reasons, suggests that the analyzed bike lanes do not serve the activities of the urban area. Therefore, some efforts should be made in this sense in order to promote urban cycling.

## Figures and Tables

**Table 1:** Sample socio-demographic and trip characteristics.

Category	Sub-Category	*n*.	%
Gender	Male	72	55.8
	Female	57	44.2
Age	Younger than 20	3	2.3
	Between 21 and 25 years old	12	9.3
	Between 26 and 30 years old	18	14.0
	Between 31 and 40 years old	38	29.5
	Between 41 and 50 years old	39	30.2
	Between 51 and 60 years old	17	13.2
	Older than 60	2	1.6
Employment status	Student	30	23.3
	Employee	53	41.1
	Freelancer	26	20.2
	Pensioner	2	1.6
	Unemployed	14	10.9
	Other	4	3.1
Marital status	Single	44	34.1
	Non-marital status	3	2.3
	Married	46	35.7
	Divorced	13	10.1
	Widower	2	1.6
	Other	21	16.3
Frequency of trip	Once a day	12	9.3
	Several times a day	8	6.2
	Once a week	59	45.7
	Once every 10 days	20	15.5
	Rarely	30	23.3
Trip purpose	Work	7	5.4
	Study	10	7.8
	Sport	97	75.2
	Shopping	15	11.6
Total		129	

**Table 2:** Cyclists’ perceptions.

Type of Perception	Path’s Aspect	Average Value
Level of comfort	Other bikes	7.02
	Shops	6.04
	Shady areas	6.49
	Temperature	6.59
	Lightening	6.36
	Odor	5.94
	Environment closed to the path	6.70
	Cleanliness	6.29
	Landscape	6.94
Degree of nuisance	Pedestrian flow	5.38
	Opposing pedestrians	5.88
	Objects obstructing the passage	4.19
	Scooters parked along the path	3.79
	Street lane width	4.56
	Street lanes number	5.16
	Traffic volume	6.14
	Trucks and buses volume	5.47
	Traffic speed	7.20
	Pollution	6.21
	Noise	6.19
Degree of protection	Theft or robbery	5.68
	Accidents on cycle path	4.91
	Accidents involving vehicles	4.55
	Weather	3.78
	Stray animals	5.09

**Table 3:** Principal component analysis.

Constructs	Items	Loadings	Eigenvalues	Variance [%]	Cronbach’s Alpha
C1: Physical Nuisance	Opposing pedestrians	0.87	7.204	28.817	0.901
	Pedestrian flow	0.85			
	Scooters parked along the path	0.78			
	Street lane width	0.77			
	Street lanes number	0.73			
	Objects obstructing the passage	0.66			
	Trucks and buses volume	0.63			
C2: Protection	Accidents involving vehicles	0.88	4.523	18.030	0.852
	Accidents on cycle path	0.85			
	Stray animals	0.75			
	Theft or robbery	0.75			
	Weather	0.65			
C3: Physical Comfort	Shady areas	0.79	3.026	12.104	0.759
	Shops	0.69			
	Temperature	0.61			
	Other bikes	0.60			
C4: Non-physical Nuisance	Pollution	0.88	1.542	6.170	0.803
	Noise	0.79			
	Traffic volume	0.59			
	Traffic speed	0.57			
C5: Non-physical Comfort	Lightening	0.81	1.147	4.588	0.787
	Cleanliness	0.80			
	Odor	0.63			
C6: Ambience	Environment closed to the path	0.79	1.038	4.151	0.853
	Landscape	0.72			

**Table 4:** Importance, performance, and gap.

Path’s Aspect	Perf.	Norm. Perf.	Imp.	Norm. Imp.	Gap (I-P)
Opposing pedestrians	7.02	0.39	0.87	0.94	0.56
Pedestrian flow	6.04	0.53	0.85	0.90	0.36
Scooters parked along the path	6.49	1.00	0.78	0.69	−0.31
Street lane width	6.59	0.78	0.77	0.63	−0.15
Street lanes number	6.36	0.60	0.73	0.52	−0.08
Objects obstructing the passage	5.94	0.88	0.66	0.28	−0.60
Trucks and buses volume	6.70	0.51	0.63	0.21	−0.29
Accidents involving vehicles	6.29	0.24	0.88	1.00	0.76
Accidents on cycle path	6.94	0.35	0.85	0.91	0.55
Stray animals	5.38	0.40	0.75	0.57	0.16
Theft or robbery	5.88	0.59	0.75	0.56	−0.03
Weather	4.19	0.00	0.65	0.27	0.27
Shady areas	3.79	0.84	0.79	0.71	−0.12
Shops	4.56	0.70	0.69	0.40	−0.30
Temperature	5.16	0.87	0.61	0.12	−0.75
Other bikes	6.14	1.00	0.60	0.11	−0.89
Pollution	5.47	0.29	0.88	1.00	0.71
Noise	7.20	0.30	0.79	0.70	0.40
Traffic volume	6.21	0.31	0.59	0.08	−0.23
Traffic speed	6.19	0.00	0.57	0.00	0.00
Lightening	5.68	0.80	0.81	0.77	−0.03
Cleanliness	4.91	0.78	0.80	0.73	−0.05
Odor	4.55	0.67	0.63	0.18	−0.48
Environment closed to the path	3.78	0.90	0.79	0.70	−0.20
Landscape	5.09	0.98	0.72	0.49	−0.48

## Data Availability

The data presented in this study are available on request from the corresponding authors. The data are not publicly available due to privacy restrictions.
